# ADME Properties in Drug Delivery

**DOI:** 10.3390/pharmaceutics17050617

**Published:** 2025-05-06

**Authors:** Luciana Scotti, Marcus Tullius Scotti

**Affiliations:** Cheminformatics Laboratory—Postgraduate Program in Natural Products and Synthetic Bioactive, Federal University of Paraíba-Campus I, João Pessoa 58051-970, Brazil; mtscotti@gmail.com

In recent decades, the identification of thousands of lead compounds, through development of analytical, synthetic, and computational techniques has occurred. However, the path followed by a bioactive compound to become a drug is long and involves differing methodologies. Different variables must be considered, such as pharmacokinetics, drug interactions, efficacy, safety, among others. The process is long and expensive.

In recent decades, absorption, distribution, metabolism, and excretion (ADME) properties turn out to be the main factors that cause the failure of bioactive compounds candidate for new drugs. The development of bioassay techniques, biotechnological methods, bio-guided phytochemical studies, automated high-throughput screening, and advanced analytical methods has revolutionized drug research. In vitro and computational models now enable researchers to analyze factors influencing the pharmacokinetics of potential drugs. While traditional in vivo studies provide precise pharmacokinetic profiles and identify issues such as dose variation, in vitro and in silico approaches offer accurate predictions, save time and resources, and mitigate ethical concerns. These methods help researchers pinpoint challenges like low solubility and poor bioavailability in orally administered drugs, as well as formulation issues. Pharmaceutical companies invest significantly in ADME screening to promptly detect undesirable pharmacokinetic profiles and refine their research accordingly.

The study of how drugs are absorbed, distributed, metabolized, and excreted in the body, has seen remarkable advancements in recent years. These developments are shaping drug discovery, personalized medicine, and therapeutic strategies:

1. Physiologically Based Pharmacokinetic (PBPK) Modeling: PBPK models have become a cornerstone in predicting drug behavior in various populations, including those with specific diseases or genetic variations. PBPK modeling is a sophisticated computational approach used to predict the absorption, distribution, metabolism, and excretion (ADME) of drugs in the human body, a transformative tool in pharmacokinetics, enabling safer, faster, and more effective drug development. By bridging complex biological systems with computational power, it stands at the forefront of precision medicine [1,2,3,4,5,6,7,8,9,10]. These models integrate physiological, biochemical, and molecular data to simulate drug interactions and optimize dosing by integrating detailed physiological, biochemical, and molecular data, PBPK modeling creates a virtual representation of the human body to simulate drug behavior under various scenarios.

In Drug Development PBPK models play a pivotal role in preclinical and clinical drug development by predicting pharmacokinetics without extensive animal testing or human trials. Regulatory agencies, such as the FDA, increasingly rely on PBPK modeling for dose optimization and to address questions related to drug safety and efficacy. The models enable individualized treatment plans by considering patient-specific factors like age, liver function, and genetic variations and helps evaluate how different drugs might interact in the body, particularly when metabolized by the same enzymes.

2. Advances Drug-Drug and Drug-Herb Interactions is in understanding how drugs interact with each other and with herbal supplements have improved safety and efficacy. Researchers are now better equipped to predict and mitigate adverse interactions, especially in complex therapeutic regimens.
3. Noncoding RNAs such as microRNAs, have been identified as key regulators of drug metabolism and transport. This discovery opens new avenues for targeting these molecules to enhance drug efficacy and reduce side effects.
4. The use of CRISPR/Cas9 technology has enabled the development of novel animal models to study drug metabolism and transport. CRISPR/Cas9 technology has opened up exciting possibilities in the field of pharmacokinetics, enabling researchers to explore drug behavior and metabolism with unprecedented precision. By using this gene-editing tool, scientists can better understand how genetic factors influence the absorption, distribution, metabolism, and excretion (ADME) of drugs [1,2,3,4,5,6,7,8,9,10].
5. Impact of Disease on Drug Metabolism has highlighted how diseases alter drug-metabolizing enzymes and transporters. This knowledge is crucial for tailoring treatments to individual patients, particularly those with chronic or progressive conditions.
6. Mathematical models are being used to simulate drug behavior under various conditions. These tools are invaluable for predicting outcomes in clinical trials and optimizing drug design.

These advancements underscore the dynamic nature of pharmacokinetics and its critical role in modern medicine. By integrating cutting-edge technologies and interdisciplinary research, pharmacokinetics continues to pave the way for safer and more effective therapies.

Future Prospects of Pharmacokinetics

The use of AI and machine learning (ML) is transforming pharmacokinetics by enabling faster and more accurate predictions of drug behavior. These technologies can analyze large datasets to identify patterns and predict pharmacokinetic parameters, enhance drug discovery and development by optimizing molecular designs, provide personalized dosing recommendations based on patient-specific factors, such as genetics and comorbidities [1,2,3,4,5,6,7,8,9,10].

PBPK modeling will continue to evolve, incorporating more complex biological systems. Future PBPK models may simulate rare and underrepresented populations in clinical trials, including pediatric or geriatric groups; pharmacokinetics of biologics and advanced therapies like monoclonal antibodies, RNA therapeutics, and gene therapies; multiscale interactions, bridging cellular-level processes to organism-level outcomes.

Pharmacogenomics, the study of how genetic variations impact drug responses, will play a central role in personalized medicine. Future advancements will identify novel genetic markers influencing drug metabolism, distribution, and transport; guiding the development of pharmacokinetic models that account for individual genetic profiles. Combined with CRISPR/Cas9 technology, they can model genetic variations or diseases to predict pharmacokinetic behavior in diverse populations.

Wearable technologies and biosensors are increasingly being used to monitor drug levels in real time. Future developments in this field as continuous tracking of pharmacokinetic parameters such as plasma concentration, real-time dose adjustments based on feedback from wearable sensors or enhanced understanding of intra-patient variability over time.

As biologics and nanomedicine continue to gain prominence, pharmacokinetics will adapt to address their unique properties. Future research may focus on understanding how nanoparticle size, shape, and coating affect drug distribution and clearance. Developing pharmacokinetic models specific to biologics like monoclonal antibodies and cell-based therapies.

The future of pharmacokinetics lies in its interdisciplinary integration with cutting-edge technologies, such as wearable biosensors, organ-on-a-chip systems, and machine learning algorithms. These innovations are paving the way for precision medicine, where therapies can be personalized to individual genetic, physiological, and environmental factors. Ethical considerations and regulatory frameworks must evolve alongside these advancements to ensure equitable access, safety, and transparency in pharmacokinetic-driven medical practices.

In summary, pharmacokinetics continues to evolve as a dynamic and essential field, advancing our capabilities to develop safer, more effective, and personalized therapeutic interventions. Its role in shaping modern medicine will undoubtedly expand, addressing the complexities of emerging therapies and global healthcare challenges.
